# Characterization and in vitro anticancer potential of exopolysaccharide extracted from a freshwater diatom *Nitzschia palea* (Kütz.) W.Sm. 1856

**DOI:** 10.1038/s41598-022-24662-z

**Published:** 2022-12-21

**Authors:** Elumalai Sanniyasi, Antony Prakash Rejoy Patrick, Kreedika Rajagopalan, Rajesh Kanna Gopal, Rajesh Damodharan

**Affiliations:** 1 Department of Biotechnology, University of Madras, Guindy Campus, Chennai, 600025 India; 2 Department of Biotechnology, College of Science and Humanities, SRM Institute of Science and Technology, Kattankulathur, Kancheepuram District, Tamil Nadu 603203 India; 3 Department of Microbiology, Saveetha Dental College and Hospitals, SIMATS, Chennai, 00077 India

**Keywords:** Biotechnology, Cancer, Plant sciences

## Abstract

Diatoms are photoautotrophic microalgae classified under class Bacillariophyceae, engulfed by hard silicate frustules, which give mechanical support and protection from bacterial infections. They exude polysaccharides extracellularly that help them with their gliding motion (locomotion). However, the bioactivity of such compounds was least explored from freshwater diatoms. In the present study, a single species of pennate diatom identified as *Nitzschia palea* was isolated and molecularly characterized by 18S rRNA smaller subunit gene (partial) sequencing and submitted to GenBank NCBI and accession number retrieved as ON360983. Based on logarithmic growth curve analysis, the exponential phase was obtained from 3rd to 4th day of diatom culture. The exopolysaccharide was extracted by the hot-water extraction method, and characterized by FT-IR. The total yield of exopolysaccharide from *Nitzschia palea* was estimated as 1.56 mg in 100 mL of culture after 7 days of incubation. The estimated carbohydrate content was 51.35 µg/100 µL. The monosaccharide constituents were determined by acid hydrolysis of exopolysaccharide, silylation (derivatization), followed by GC–MS analysis and tabulated. The extracted exopolysaccharide was evaluated for its anti-cancer potential against the Human Adenocarcinoma lung cancer cell line (A549) and the estimated IC_50_ value was 62.64 µg/mL. Acridine orange staining assay and DNA fragmentation assay also confirmed the apoptotic activity of exopolysaccharide derived from the diatom *Nitzschia palea*.

## Introduction

Cancer caused around 9.6 million deaths in the year 2018, most of which occurs due to metastases. Lung cancer is more common in males and primarily occurs due to DNA damage, abnormal repair mechanisms, and damaged tumor suppressor gene. Bioactive compounds from nature have several beneficial effects in treating cancer than synthetic drugs, but the exploration of such compounds from some microorganisms is completely unexplored. Exopolysaccharides (EPS) are heterogenous carbohydrate polymers bound around and give a slimy texture to the cells reported in microalgae, bacteria, yeast, and fungi^1^. EPS is vastly used as a food thickening agent, emulsions in the cosmetic sector, pharmaceuticals, flocculants, additives, preservatives, and petroleum industries^2,3^.

A probiotic bacterium *Bacillus albus* DM-15 showed potent anti-proliferative activity in human lung cancer cells (A549) in vitro^1^. In another interesting study, EPS derived from a bacterium *Bacillus subtilis* isolated from a housefly was reported to inhibit the proliferation of cervical cancer cells (HeLa)^4^. EPS from a lactic acid bacterium also suppressed the tumor progression and promotes apoptotic cell cycle arrest with promising anticancer activity^5^. Simultaneously, in an in vivo *study*, the EPS from *Lactobacillus plantarum* NCU116 inhibits the growth of the tumor and induces apoptosis by enhancing the expression of Fas, Fasl, and c-Jun with a potent anticancer activity^6^. Concurrently, the EPS from *Lactobacillus helveticus* MB2-1 also showed potent anticancer activity against human colon cancer cells (HT-29) through the process of apoptosis^7^. The EPS from *Rhodococcus erythropolis* HX-2 rich in monosaccharide units like fucose, glucose, galactose, glucuronic acid, and mannose had shown anticancer activity on lung cancer cells (A549), liver cancer cells (SMMC-7721), and cervical cancer cells (HeLa)^8^. However, EPS derived from marine bacteria such as *Brevundimonas subvibrioides, Bacillus thuringiensis, B. amyloliquefaciens, Pseudomonas fluorescens*, and *Advenella kashmirensis* constitute sulfated groups in their monosaccharide units and had shown anti-proliferative activity on liver cancer cells HepG2^9^. An EPS from a thermophilic bacterium *Anoxybacillus gonensis* YK25 isolated from the hot springs of Turkey hampered the proliferation rate of tumor progression in metastatic bone tumors (SHSY5Y), human colon cancer (HT29), prostate cancer (DU145), and human lung cancer A549^10^. Bacterial EPS was reported with anticancer potential, whereas, EPS from diatoms (Bacillariophyceae) is unexplored.

Diatoms are an ideal source of bioactive compounds due to the wide range of secondary metabolites synthesized by them within a short life span. Acyl glycerides, polysaccharides, oxylipins, and fatty acid esters are the major class of potential anticancer compounds isolated from marine diatoms. The compounds which can be extracted from living organisms have a great impact on the infection and are less toxic to the human response. Diatoms exudate bioactive compounds like polysaccharides, fucoxanthin, and polyunsaturated fatty acids. The growth conditions of a microalga may also have an impact on its bioactivity. For instance, the anti-cancer property of diatom extracts has been found to vary when they were cultured in different temperature and light conditions^11^.

Diatoms are predominant among the primary producers in the aquatic ecosystem stabilizing the global carbon cycle and are cosmopolitan in nature including the Arctic and Antarctic. They synthesize their frustules from a very minute amount of silica present in water and thus play an engrossing role in the field of Nanotechnology. The frustules make the cell wall of the diatoms like an envelope over the organic content of the diatomaceous cell. Commonly, the diatoms release the organic content out of the frustule when it is expired and the frustule remains intact as fossil sediments sequester nitrogen and other constituents from the aquatic ecosystem^12^.

Generally, diatoms are classified based on the shape of the frustule into two groups namely Pennate and Centric diatoms. Pennate diatoms are bilaterally symmetrical, whereas, centric diatoms are radially symmetrical^13^. Morphological identification of diatom is based on the characteristic features of its frustule including the number of Raphe, Striae, and Fibulae^14^. The raphe is a slit present on the frustules of some diatoms responsible for locomotion. Stria is a row of areolae, or alveoli, in the plural form it is called striae. Fibulae is a plural of Fibula; it is an internal bar that supports the raphae slit. However, molecular characterization using 18S rRNA gene sequencing would be fruitful to identify isolated diatom cultures.

Exopolysaccharides are secreted as a slimy texture by the diatoms for their gliding motion (locomotion) and adherence to the surface^15^. Therefore, the motility of the diatoms depends on the mucus exopolysaccharide production, which is secreted and released by raphe^16,17^. The exopolysaccharide is most commonly heteropolysaccharide with uronic acid, and sulfate groups of significant content^18–20^.

Even though diatoms are single-celled eukaryotic organisms, their life cycle ends within 24 h. They are usually asexual means of reproduction similar to binary fission and thus have a slightly faster growth rate^21^. External parameters like chemical and physical factors may affect their growth either at a higher rate in the form of bloom or by inhibiting the growth of diatoms. When they are provided with suitable temperature, light, and nutritive medium they can survive for a longer period. When compared with other organisms the life cycle is too short and effective in the production of polysaccharides.

In this study, a pure culture of freshwater diatom identified as *Nitzschia palea* was isolated from an inland water reservoir. The exopolysaccharide extracted from the diatom was characterized and evaluated for its anticancer potential on Human Adenocarcinoma Lung Cancer (A549) cells in vitro.

## Materials and methods

### Sample collection

The freshwater sample was collected from Chembarambakkam Lake of Kanchipuram District, Tamil Nadu, India. About, 1 L of water sample was collected from the lake and brought to the laboratory followed by the analysis of physicochemical parameters of water by YSI 650 MDS water analysis instrument.

### Isolation of diatom

The collected freshwater sample was subjected to filtration with a nylon mesh to remove debris from the water. About 20 mL of water sample was centrifuged at 3000 rpm for 5 min. in a cooling centrifuge (Remi C-24 plus). The obtained pellet was diluted in 2 mL of distilled water and vortexed. Then, 50 µL of the sample was observed under the light microscope (Lawrence and Mayo equipped with ScopeImage 9.0), and microphotographs were recorded. For the isolation of diatom, about 1 mL of the concentrated water sample was used as an inoculum in an autoclaved 100 mL of PM culture broth medium^22^ (with pH 6.0) in 250 mL of the conical flask. After inoculation, the flask was kept under incubation for two weeks at 12 h of light and dark respectively in an Algal culture room from 18 to 20 °C. Then, about 100 µL of broth culture grown in a 250 mL Conical flask was taken as inoculum for the spread plate technique in a Petri dish with 20 mL of PM medium solidified with 2.5% Agar. Again the plates were kept for incubation under 12 h of light and dark conditions for two weeks in the Algal culture room at 18–20 °C.

A distinct diatom colony growing on the spread plate was further transferred (loop full pull of inoculum) to a fresh Petri dish with PM solidified culture medium using quadrant streak plate technique for obtaining a pure culture of diatom and incubated at the same conditions discussed above for two weeks. Then the pure diatom culture was introduced into a PM broth culture medium, sub-cultured, and maintained in the Algal culture room.

### Morphological identification of diatom

The morphological identification of diatom was based on the morphological structure and features of frustules. The isolated pure diatom culture was identified based on unique morphological features including the presence of raphae, the number of raphae, length, and the number of striae, and fibulae found on the frustules of the diatom.

#### Acid digestion of organic matter

For morphological identification of the diatom, the organic content present in the diatom should be removed to obtain clear frustules. About 10 mL of the diatom broth culture was subjected to centrifugation at 1000 rpm for 3 min. to yield a pellet of diatom without breaking the frustules (high rpm may brittle frustule). The pellet was then treated with acetic acid and the sulphuric acid solution mixed at a ratio of 9:1 for a duration of 30 min in a boiling water bath (90 °C). After successful acid digestion of organic matter, the content was again centrifuged at 3000 rpm for 3 min. Then other debris found in the pellet was washed with 2 mL of ethanol and again centrifuged at 1000 rpm for 2 min. Then the obtained pellet containing frustules devoid of organic matter was diluted in 1 mL of distilled water, observed under a light microscope, and subjected to Scanning Electron Microscopic (SEM) analysis.

#### Scanning electron microscope (SEM) imaging

The acid-digested frustules were fixed on a glass slide covered with aluminum foil and air-dried at room temperature. Then coated with chromium in an ion sputter and observed under SEM (Theromofischer Apreo S Scanning electron microscope) and imaged at 6500X to 25000X magnification.

### Molecular characterization of *Nitzschia palea*

#### Isolation of genomic DNA

A 100 mL of 14 days old pure culture of *Nitzschia palea* was centrifuged at 2000 rpm for 5 min. to obtain a cell pellet. Then the cell pellet was dispersed in 1 mL of Milli-Q water and sonicated at 40 kHz for 10 min. using an ultrasonicator to break the frustules and get rid of the cell content. About 1 mL of Lysis buffer (4 M Urea, 0.2 M Tris–HCL, 20 mM NaCl, 0.2 mM Na_2_EDTA, pH of 7.4) and 100 µL of Proteinase-K (20 mg/mL) was added to the cell pellet and kept for incubation at 45 °C for 1 h. Then 1 mL of preheated (40 °C) DNA extraction buffer (3% Cetyl trimethyl ammonium bromide (CTAB), 1.4 M NaCl, 20 mM Na_2_EDTA, 0.1 M of Tris–HCl, 1% β-mercaptoethanol) was added and again incubated at 40 °C for 1 h. Then twice the volume of chloroform and isoamyl alcohol solution in the ratio of 24:1 was added and mixed thoroughly. After thorough vortexing, the content was centrifuged at 13,000 rpm for 5 min. and the aqueous phase was conserved in fresh microcentrifuge tubes. For precipitating DNA, twice the volume of 99.99% ethanol was added to the collected aqueous phase, and the tube was incubated at −20 °C for 60 min. Then the precipitated DNA content was subjected to centrifugation at 13,000 rpm for 5 min., and obtained DNA pellet was washed thrice with 70% ethanol and air-dried to be devoid of excess ethanol. Finally, the isolated DNA was diluted in 100 µL of TE buffer and stored at −20 °C. The concentration and purity of the isolated DNA were determined by Biophotometer D30 plus (Eppendorf), and TE buffer was used as blank. The absorbance was measured at 260 nm and the purity of DNA was determined by A260/280 absorbance ratio.

#### Agarose gel electrophoresis

Agarose gel electrophoresis was carried out and the quality of the isolated DNA was checked using 1% agarose gel and ethidium bromide (EtBr) (30 µL) (1 mg/mL) was used as a stain. About 0.5 X TAE buffer was used as tank buffer, and the DNA sample (30 µL) was mixed with the gel loading dye (390 µL of glycerol, 2.5 mg bromophenol blue, 50 µL of 10% SDS, and 20 µL of EDTA) and loaded into the wells. Then the gel was electrophoresed at 100 V at room temperature for 20 min. For visualization of the isolated DNA bands, the gel was trans-illuminated under UV light and photographed using Vilber Bio-print by Eppendorf.

#### PCR amplification of 18S rRNA gene

The PCR mix constitutes 1 µL (61 ng/µL) of isolated DNA template, 1 µL of forward and reverses primers each (10 pM/µL) (Table 1), 2 µL of 10 mM deoxynucleotide mix, 5 µL of 10X PCR buffer (25 mM MgCl_2_ buffer) and 1 µL of *Taq* DNA polymerase (5U/µL). Then the total mix was made up to 50 µL with PCR-grade water. PCR amplification consists of 34 cycles, each cycle with denaturation 94 °C for 45 s, annealing 62 °C for 60 s, and elongation 72 °C for 2 min. However, initial denaturation was extended up to 2 min. before starting denaturation initially, whereas, elongation was also extended after the completion of PCR cycles for 10 min. Then the amplified PCR products were qualified using 1% agarose gel with EtBr as a dye and observed under UV transillumination and photographed. Sangers method of dideoxy sequencing was employed in this study to obtain 18S rRNA sequence amplified from the DNA template of *Nitzschia palea* using a 3500 Genetic analyzer (Applied Biosystems). From the obtained forward and reverse sequences, a contig sequence (18S rRNA partial gene) was generated by using DNA Baser Assembler v5.15.0.

**Table 1:** 18S rRNA primer used for Molecular characterization of *Nitzschia palea.*

Primer used	Nucleotides
P73 – Forward Primer	5 ’ AAT CAG TTA TAG TTT ATT TGR TGG TACC 3 ’
P47 – Reverse Primer	5 ’ TCT CAG GCT CCC TCT CCG GA 3 ’

#### Phylogenetic analysis

The 18S rRNA partial gene of *Nitzschia palea* sequenced was subjected to Nucleotide BLAST in NCBI, and similar sequences aligned with high hits were retrieved from NCBI. Then the retrieved sequences were further aligned pair-wise, and multiple-sequence by ClustalW sequence alignment with the 18S rRNA gene of *Nitzschia palea* using the MEGA X package. Simultaneously, the 18S rRNA gene of *Nitzschia palea* was submitted to GenBank, NCBI (National Centre for Biotechnology Information), and the accession number was retrieved. The phylogenetic tree was constructed using the MEGA X package (Molecular Evolutionary Genetics Analysis X). The neighbor-joining statistical method was employed to construct the phylogenetic tree with 500 numbers of bootstrap replications, gamma distributed, and pairwise deletion with a sum of branch length (SBL) value of 0.63.

### Logarithmic growth curve

To study the logarithmic growth curve, 100 mL of PM culture broth was prepared in 250 mL of a glass conical flask and inoculated with 1% inoculum. The logarithmic growth rate of *Nitzschia palea* was determined by enumerating the number of cells in each quadrant of the Haemocytometer using a light microscope every 24 h of intervals for 14 days. About 50 µL of culture was placed on a haemocytometer and observed under the light microscope under 4X magnification. The number of cells in each quadrant was enumerated for four quadrants, and the average number of cells was noted every day for a period of 14 days. Then a logarithmic growth curve was plotted in a graph and resulted.

### Extraction of exopolysaccharide

The exopolysaccharide was extracted from the diatom *Nitzschia palea* cultured in 400 mL of PM broth inoculated and incubated for six days under 12:12 h of light and dark conditions at room temperature in an algal culture room. The exopolysaccharide synthesized extracellularly by the diatom was extracted by the hot-water extraction method. The 400 mL of culture broth was filtered through Whatman No.1 filter paper to obtain cell-free aqueous filtrate, and it was kept in a water bath for 70 °C for 40 min. Simultaneously, twice the volume of absolute ethanol was added to the heat-treated aqueous filtrate and kept for incubation at −20 °C for overnight to enhance the precipitation. Then the precipitated exopolysaccharide was obtained in pellet by the centrifugation at 8000 rpm for 5 min. The pellet was dissolved in 1 mL of Milli-Q water and kept in a hot mantle at 10 °C to obtain a dry yield.

### Characterization of Exopolysaccharide

#### Estimation of Carbohydrate

The total carbohydrate content was determined from the extracted exopolysaccharide of Diatom *Nitzschia palea* by Dubois et al.^23^ Phenol-Sulphuric acid method with dextrose as standard.

#### Fourier Transform Infra-Red FT-IR analysis of Exopolysaccharide

About 1 mg of the dried exopolysaccharide sample obtained from *Nitzschia palea* was blended with potassium bromide (KBr) and compressed into a small tablet. Then the sample was analyzed for FT-IR (Broker α-E (ATR, Lab India Instruments Pvt. Ltd., Mumbai)) frequency range between 500 and 4000 cm^−1^.

#### Acid hydrolysis of exopolysaccharide and Thin Layer Chromatography Analysis (TLC)

About 10 mg of the exopolysaccharide isolated from *Nitzschia palea* was diluted in 1 mL of Milli-Q water in a microcentrifuge tube. Then 500 µL of 10% HCl was added and boiled at 80 °C for 1 h. Once acid hydrolysis was carried out, TLC was performed using a silica gel plate as the stationary phase, and the mobile phase was a mixture of chloroform, acetic acid, and water in a ratio of 6:7:1. The acid hydrolyzed sample was spotted on the gel plate using a capillary tube and TLC was carried out. After performing the TLC, the plate was removed from the coupling jar and sprayed with a 5% sulphuric acid solution (dissolved in ethanol). Once the spray was dried, the gel plate was heated at 50 °C to obtain a clear spot.

#### Gas Chromatography–Mass Spectrometry (GC−MS) analysis of Monosaccharide composition of an exopolysaccharide isolated from *Nitzschia palea*

About 500 µL of acid-hydrolyzed exopolysaccharide sample was subjected to derivatized with 500 µL of a mixture of pyridine, hexamethyldisilazane, and trimethylchlorosilane in a ratio of 9:3:1 in a watch glass. Then the mixture was subjected to GC–MS analysis using a Gas chromatograph Agilent 7890B, with HP- 5MS (5% Phenyl methyl siloxane-) column of size 30 m × 250 µm × 0.25 µm connected to a 5977A mass-spectrometry detector. A 1 µL of the sample was injected at a temperature of 250 °C and the detector was kept at 280 °C. The initial temperature of the column was set at 60 °C for 1 min and was gradually increased to 325 °C and was maintained for 10 min. Helium was used as the carrier gas at a flow rate of 1 mL/minute. The split ratio was kept as 1:1. The compounds detected by the detector were identified by comparing them to the data present in the National Institute of Standards and Technology MS2011 library.

### Anticancer potential of Exopolysaccharide isolated from *Nitzschia palea*

#### MTT Cytotoxicity Assay

In this colorimetric assay, the metabolic activity of live cells was determined by converting MTT [3-(4,5- dimethylthiazol-2-yl)-2,5-diphenyltetrazolium bromide] tetrazolium dye to purple-colored formazan crystals by the reduction of NADPH dependent oxidoreductase enzyme inside the mitochondria of living cells. A similar kind of reaction would not happen if cell death occurs and MTT dye remains idle. For assessing the anticancer activity of the exopolysaccharide isolated from *Nitzschia palea*, Human Lung Adenocarcinoma Cancer cell line A549 was employed. First, the cell line was cultured in 96-well plates (1 × 10^6^ cells/well) for 24 h in a modified DMEM culture medium. Then the cell cultures were again incubated for 24 h after replacing the spent medium supplemented with the test sample (diatom exopolysaccharide) at different concentration aliquots between 250 µg/mL to 15 µg/mL. The cells in the wells were washed with phosphate-buffered saline (pH 7.4) and 0.02 mL of MTT (5 mg/mL in PBS) was added and incubated for 4 h at 37 °C in a CO_2_ incubator. After the removal of the remaining medium from the wells, 100 µL of DMSO was provided and incubated for 10 min. Finally, the optical density (OD) was determined for each sample using a microplate reader at 570 nm (Thermo Multiskan EX, USA). Untreated cells were used as control. Bright-field microscope (40X magnification) was used to image the morphology of the control and the treated cells after 24 h. Cell viability was determined by the equation given below. The test sample with respective concentrations was evaluated in triplets, and the average percentage of inhibition with the standard error was calculated and interpreted. The IC_50_ value of the given test sample was calculated using the graph plotted.

$${\text{Cell viability }}\left( {\text{in percent}} \right) \, = \, \left( {{\text{OD of treated cells }}/{\text{ OD of untreated cells}}} \right){\text{ x 1}}00$$

#### Acridine orange staining assay

The acridine orange stains with membrane disrupted dead cells and observed orange in color, whereas, live cells were observed green in color in a fluorescent microscope. Hence, the apoptotic dead cells incorporated an acridine orange stain. Similarly, necrotic cells also stain orange color but are devoid of condensed chromatin and resemble the morphology of living cells. Both the control and test sample (Diatom exopolysaccharide) treated human adenocarcinoma cancer cells for 24 h of incubation were fixed in a glass slide with methanol and glacial acetic acid (3:1 ratio) for 30 min, washed with PBS buffer, and stained using acridine orange staining solution. Again washed with PBS buffer the slide was viewed under the fluorescent microscope and photographed at 40 × magnification.

#### DNA fragmentation assay

The DNA fragmentation assay reveals the differentiation between apoptotic cell death from live cells. The control and test sample (diatom exopolysaccharide) treated cells (1 × 10^6^ cells/well) were suspended in 10 mL of Tris–EDTA buffer (10 mM Tris HCl, and 10 mM of EDTA with Ph 8.0). Proteinase K was added (20 mg of Proteinase in 1 mL of Tris–EDTA with 2% SDS) and incubated at 37 °C for 3 h. Then treated with alcohol solution constitutes phenol, chloroform, and isoamyl alcohol (25:24:1) and then DNase-free RNase (20 mg/mL) was added and incubated for 45 min. at 4 °C. To the aqueous phase, twice the volume of ethanol was added with 1 mL of sodium acetate added (2.5 M). After washing thrice with four volumes of absolute ethanol, the isolated DNA of control, and test samples treated were electrophoresed in 2% agarose gel and EtBr was used as a dye and visualized under UV Trans-illumination and photographed. The DNA fragmentation assay in agarose gel differentiates live and apoptotic cells, in which, a single DNA band shows live cells, whereas, dragged DNA bands like a ladder represents the cells undergoing apoptosis. The fragmentation of DNA is due to the activation of caspase-activated DNase (CAD) which leads to apoptotic cancer cell death.

## Results

### Collection of freshwater sample

**Figure 1:**
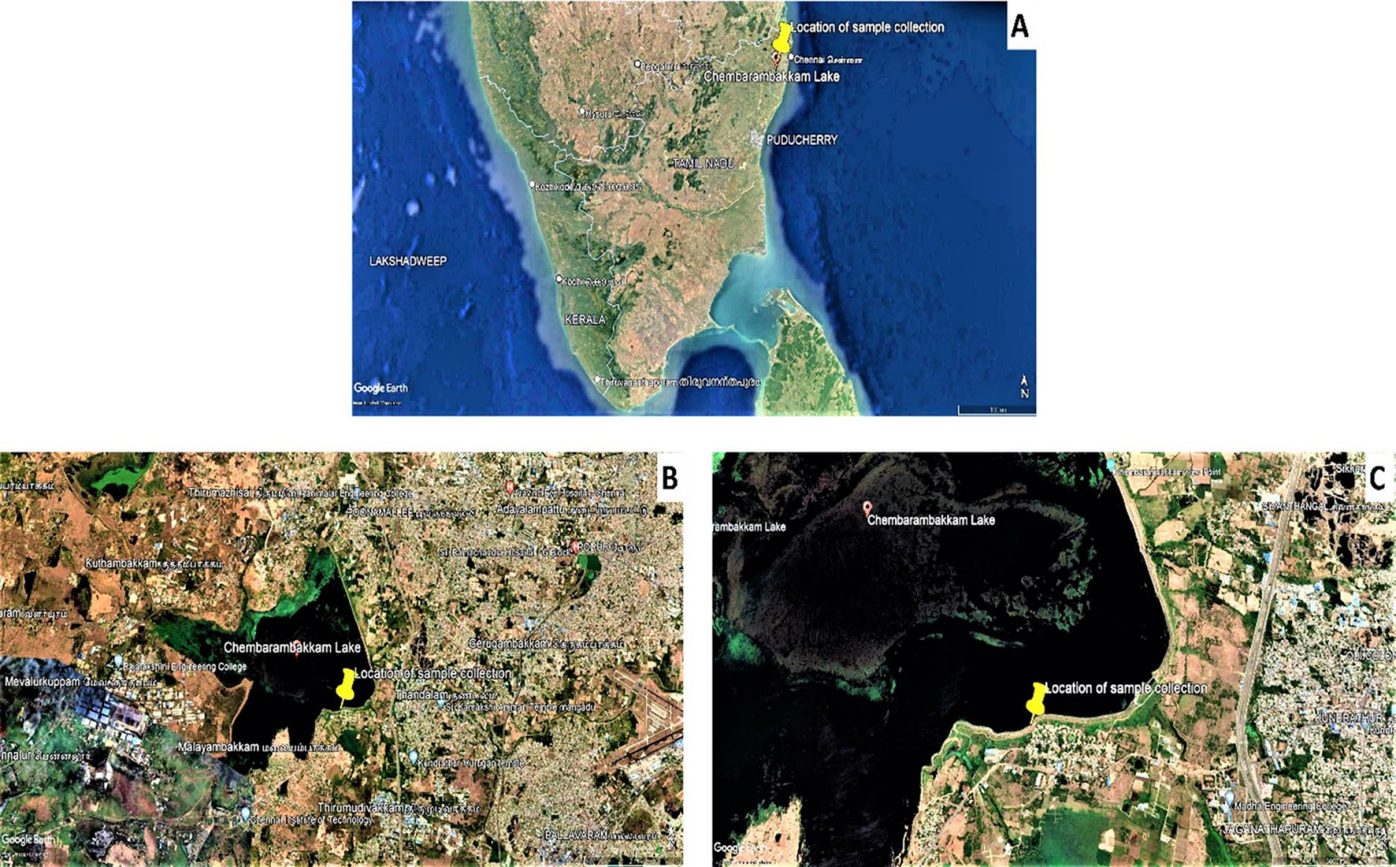
(**A**) Topogeographical image showing Southern part of India, in which the sample collection site located at the Top of the Northeastern Tamil Nadu, near Chennai City; (**B**) The image showing a clear view of the outline of the Chembarambakkam Lake; (**C**) The image showing the exact location of water sample collection (Retrieved from Google Earth Pro 7.3.4.8573 (https://google-earth-pro.updatestar.com/en)).

The freshwater sample was collected from the inland Chembarambakkam Lake of Kanchipuram District, Tamil Nadu, India which is geographically located at a latitude of 12^o^59′50.67″ N, and longitude of 80^o^4′20.96″ E (Fig. 1) (Google Earth Pro 7.3.3.7699).

### Physicochemical analysis of Water sample

The conductivity of the collected water sample was 0.147 MS/cm with a specific conductance of 0.141 MS/cm^c^, and the resistivity of the water was high with 6797.66 Ω Cm with a low TDS of 0.09 g/L. The salinity of the water sample was also low (0.07 ppt (parts per thousand)) with a high dissolved oxygen content of 15.42 mg/L, which was 194%, and 46.1 by charge. The pH of the water sample was 6.1, which was 13.7 mV, whereas, the oxidation–reduction potential was 58.3 ORP (Table 2).

**Table 2:** Physicochemical parameters observed from the freshwater sample collected from Chembarambakkam lake on 3rd January 2022.

S. No	Physicochemical parameters	Value	Unit
1	Specific Conductance	0.141	Ms/Cm^c^
2	Conductivity	0.147	Ms/Cm
3	Resistivity	6797.66	Ω Cm
4	Total Dissolved Solids (TDS)	0.09	g/L
5	Salinity	0.07	Sal
6	Dissolved Oxygen (%)	194	%
7	Dissolved Oxygen (mg/L)	15.42	Mg/L
8	Dissolved (Ch)	46.1	Ch
9	Potential of Hydrogen	6.1	pH
10	Potential of Hydrogen	13.7	pH mV
11	Oxidation Reduction potential	58.3	ORP

### Microscopic observation of water sample

**Figure 2:**
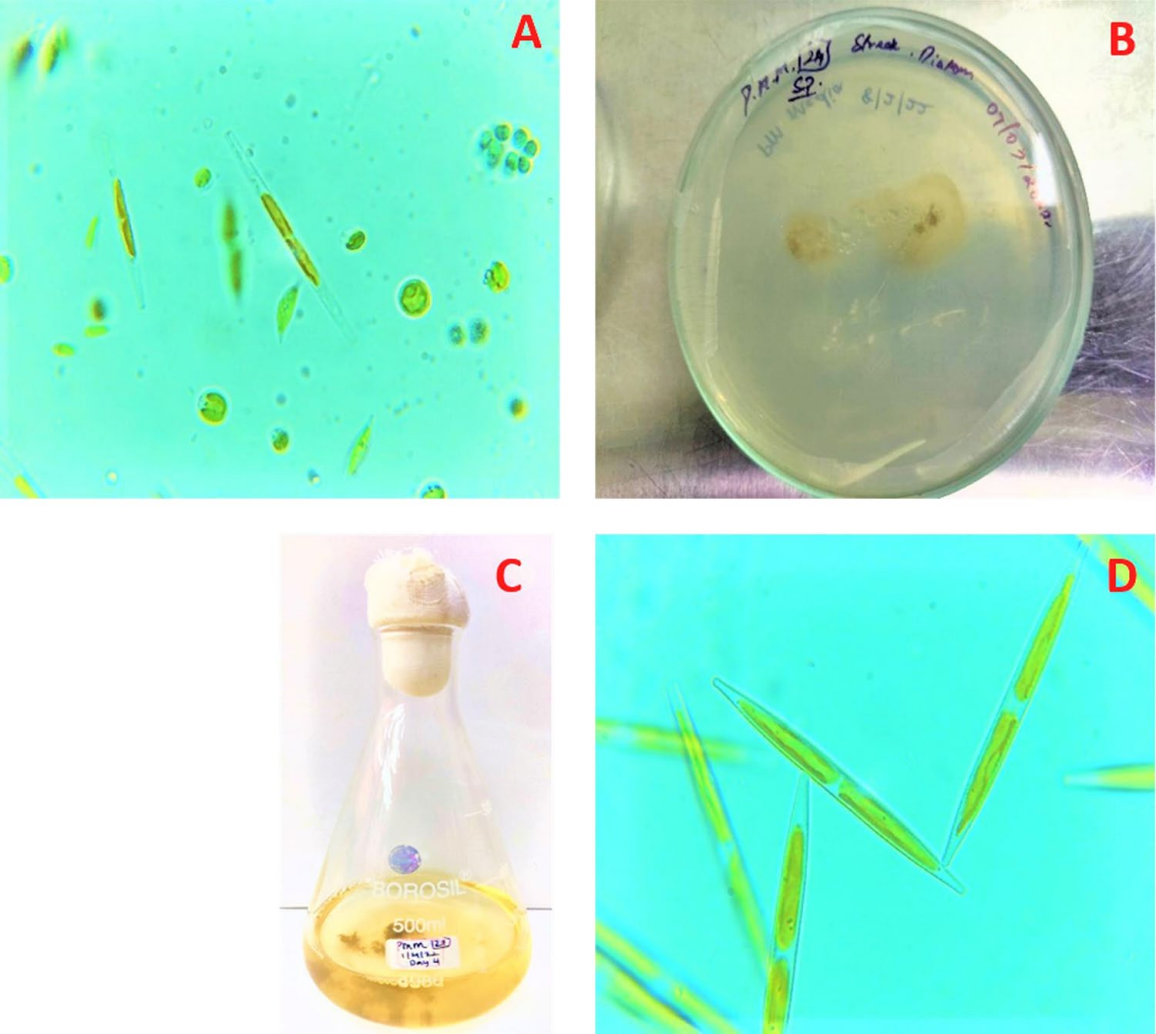
(**A**) Microscopic observation of freshwater sample collected from Chembarambakkam Lake (10 X magnification); (**B**) Spread plate technique showing diatom colony; (**C**) Isolated pure culture of Diatom *Nitzschia* sp. in PM culture broth medium (**D**) Isolated pure Diatom *Nitzschia* sp. (40 X Magnification).

The microscopic observation of water samples collected from Chembarambakkam inland lake showed microalgal diversity of diatom (Bacillariophyceae) *Nitzschia* sp., cyanobacterium (Cyanophyceae) *Merismopedia* sp., and Green alga (Chlorophyceae) *Chlorella* sp., and *Scenedesmus* sp. (Fig. 2A). Pure culture of diatom *Nitzschia* sp. was isolated from the Chembarambakkam Lake and sub-cultured in the Algal Culture Laboratory (Fig. 2B–D).

### Morphological identification of diatom

**Figure 3:**
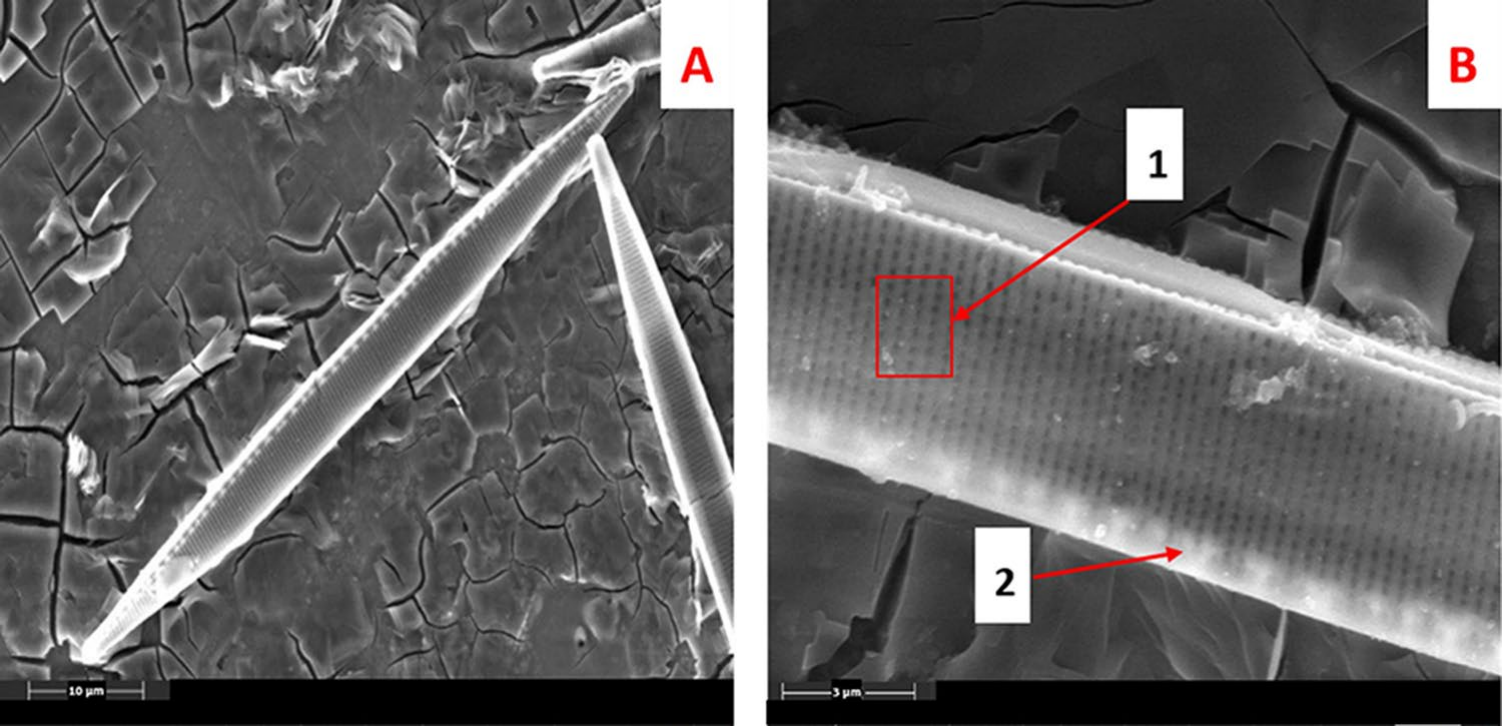
(**A**) SEM image of acid digested cell of Diatom *Nitzschia* sp. showing its frustule; (**B**) Magnified view of frustule surface showing it characteristic feature Striae, and Fibulae marked as 1 and 2 respectively.

For morphological identification of diatoms, frustule was isolated by acid-digestion of organic matter. The frustule of the diatom *Nitzschia* sp. was morphologically identified based on the characteristic features such as the isolated diatom structure pennate shape, and raphid with a length of 60 µm. The presence of 30 striae and 10 fibulae were evident, each of 10 µm in length (Fig. 3). Hence, based on the unique morphological features, the isolated diatom was morphologically identified as *Nitzschia palea*.

### Molecular characterization of *Nitzschia palea*

**Figure 4:**
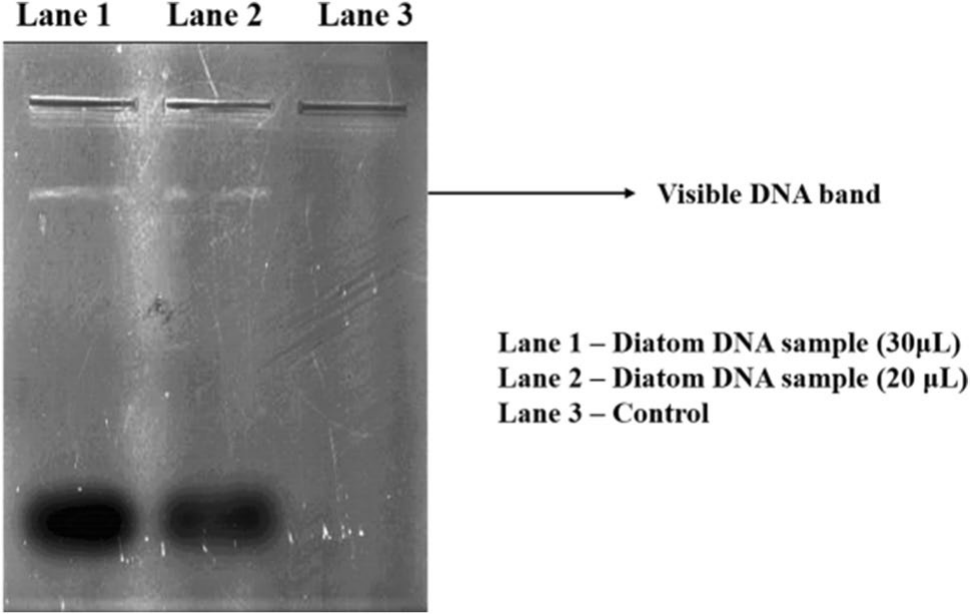
Agarose gel showing isolated genomic DNA of *Nitzschia palea* in Lane 1 and 2.

**Figure 5:**
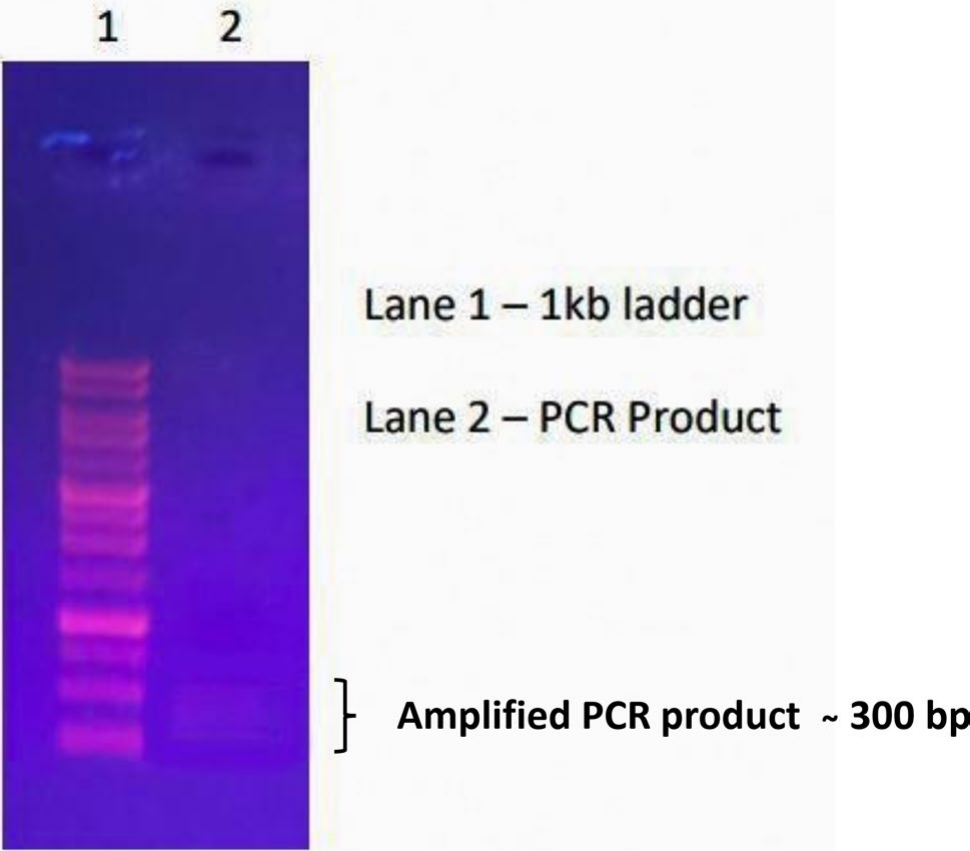
PCR amplified 18S rRNA gene smaller subunit partial sequence of the diatom *Nitzschia palea* at lane 2.

**Figure 6:**
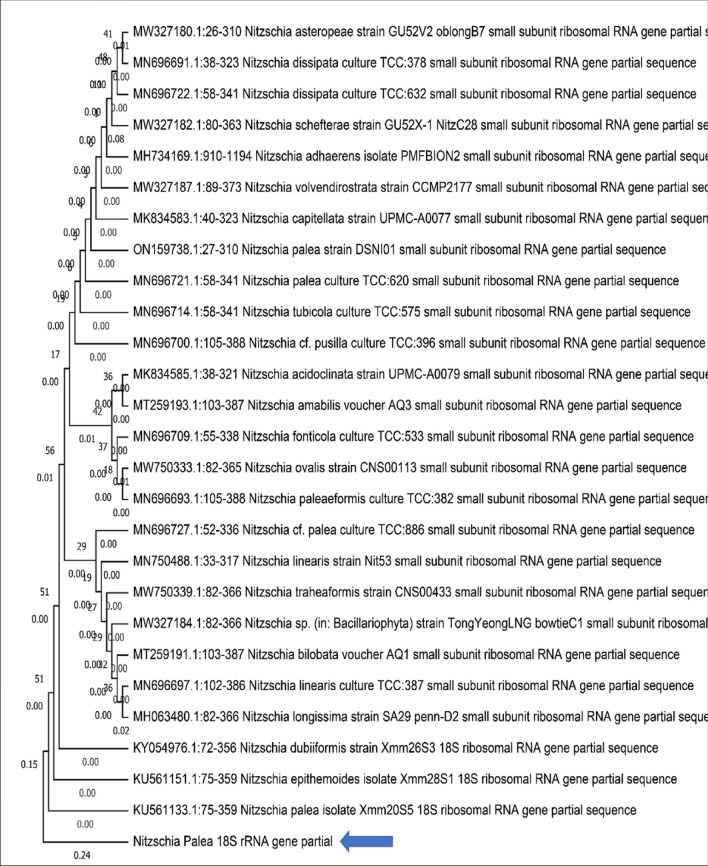
Phylogenetic tree constructed for 18S rRNA gene smaller subunit (denoted in Blue Arrow) based on Neighbour-joining statistical method.

The genomic DNA isolated from the pure culture of *Nitzschia palea* showed pure, single, and visible genomic DNA bands in agarose gel (Fig. 4) and the estimated concentration of genomic DNA was 61 µg/mL. The 18S rRNA smaller subunit partial gene of *Nitzschia palea* was amplified and the PCR product was qualitatively analyzed by agarose gel electrophoresis (Fig. 5). Based on the neighbour-joining phylogenetic tree, the 18S rRNA smaller subunit partial gene of the isolated diatom *Nitzschia palea* was closely related to the 18S rRNA partial gene of *Nitzschia palea* accession Number: KU561133 (Fig. 6). Hence, based on the molecular characterization, the isolated diatom was identified as *Nitzschia palea.* The 18S rRNA smaller subunit partial gene of the isolated diatom *Nitzschia palea* constitutes 285 bp and was submitted to GenBank and the Accession number retrieved as ON360983 (https://www.ncbi.nlm.nih.gov/nuccore/ON360983.1/**).**

### Logarithmic growth curve

**Figure 7:**
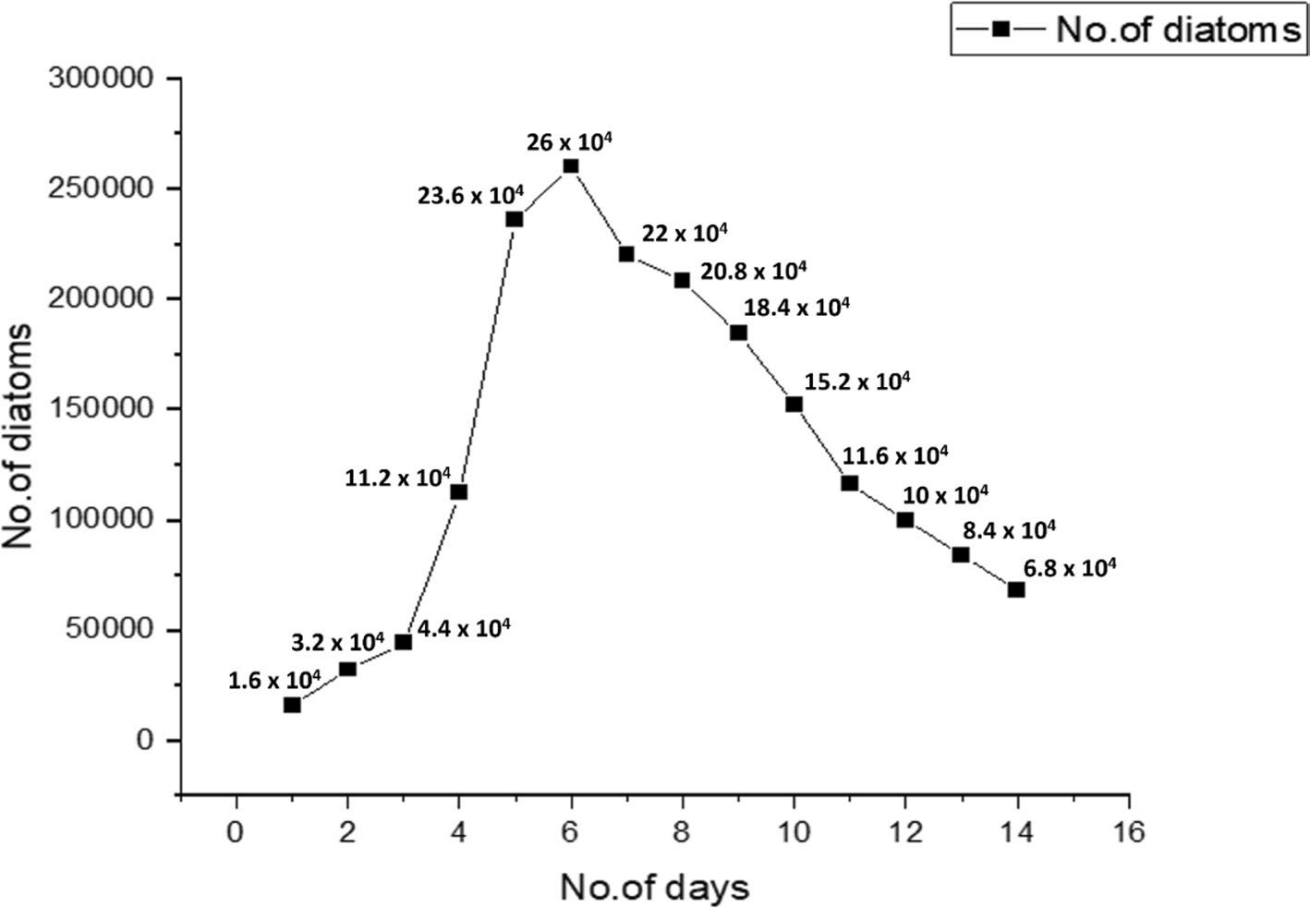
Logarithmic growth curve analysis of Diatom *Nitzschia palea* for 14 days, cultured in 100 mL of PM culture broth.

Binary cell division is the only mode of asexual reproduction in this diatom *Nitzschia palea* within 24 h. Based on the haemocytometer cell count study, the diatom starts initiating its exponential phase from 72 h (3rd day) of inoculation until 120 h (5th day) in the PM culture broth medium. However, the growth gradually declines after 144 h (6th day) of culturing (Fig. 7). Therefore, the 4th and 5th day of diatom culture at its exponential phase is preferably good for the sub-culturing of this diatom.

### Extraction of exopolysaccharide

The total exopolysaccharide content from 7-day-old diatom *Nitzschia palea* culture in 100 mL of PM broth medium was about 1.56 mg of dry weight.

### Estimation of carbohydrate

The estimated carbohydrate content of the exopolysaccharide isolated from the diatom *Nitzschia palea* was 51.35 µg/100 µL.

### Fourier transform infra-red FT-IR analysis of exopolysaccharide

**Figure 8:**
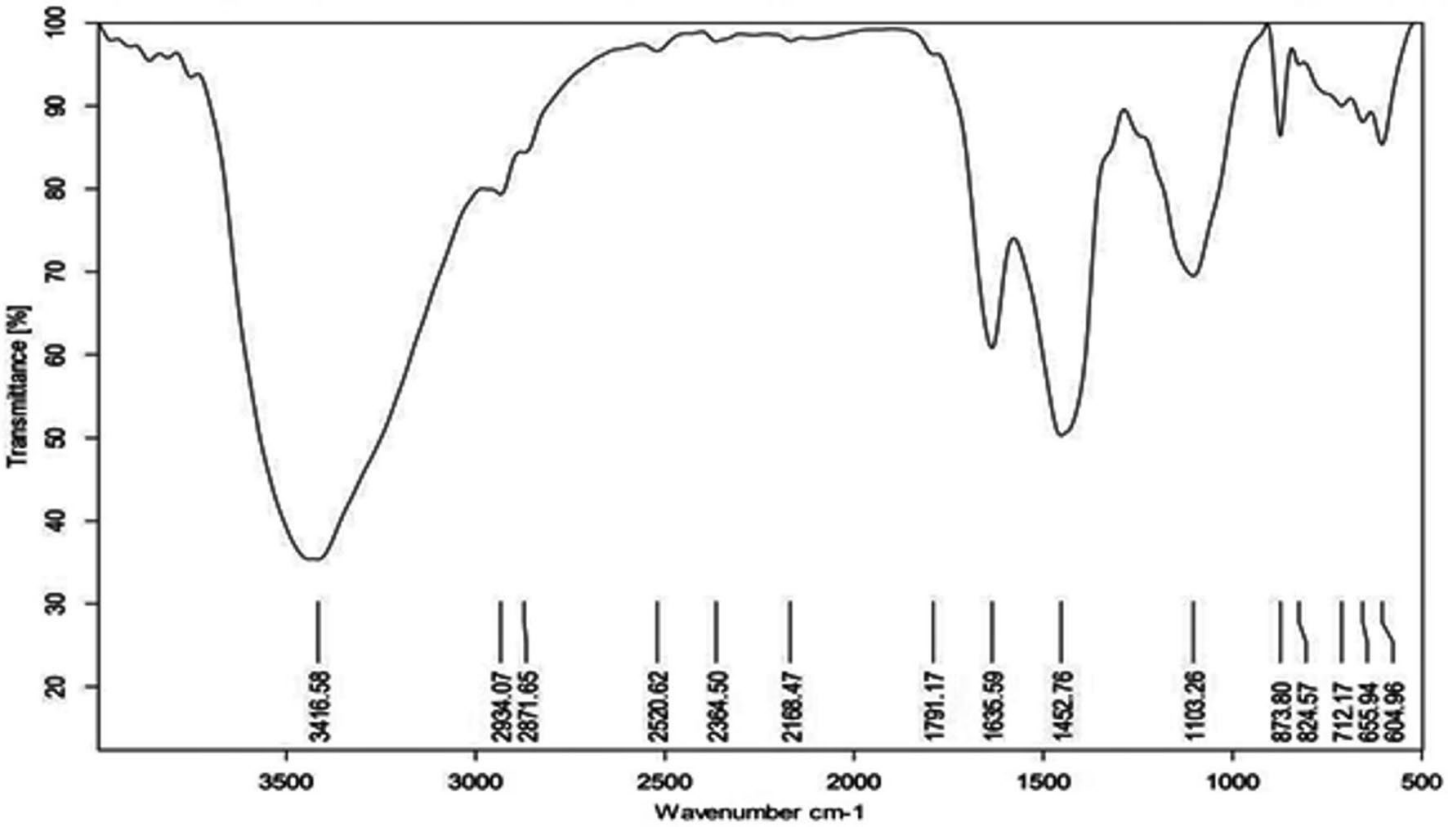
FT-IR spectrum of exopolysaccharide isolated from the Diatom *Nitzschia palea.*

The hydroxyl groups were determined based on the peak value at 3416.58 cm^−1^. Similarly, weak peak at 2520. 62 cm^−1^ represents the carboxylic acid group, whereas, IR vibrations at 2934.07 cm^−1^, 2871.65 cm^−1^, and 873.80 cm^−1^ denote the presence of C-H bond stretching of Alkane and Aromatic groups respectively. However, the peak value at 2168.47 cm^−1^, 1635.59 cm^−1^, and 1452.76 cm^−1^ link to alkynes, alkenes, and single-bonded aromatic rings respectively. Finally, the IR transmittance inhibition at 1103.26 cm^−1^ correlates with the occurrence of carboxylic acids (Fig. 8 and Table 3). Therefore, it is very clear that the functional groups of the exopolysaccharide isolated from the diatom *Nitzschia palea* correspond to the carbohydrate content.

**Table 3:** Table showing FT-IR peak values represents the functional groups of exopolysaccharide isolated from *Nitzschia palea.*

S. No	Frequency range (cm^−1^)	Functional group
1	3416.58	OH (H–bonded)
2	2934.07	C–H
3	2871.65	C–H
4	2520.62	O–H
5	2168.47	C≡C
6	1791.17	C = O
7	1635.59	C = C
8	1452.76	C–C (in ring)
9	1103.26	C–O
10	873.80	C–H

#### Thin layer chromatography analysis (TLC)

**Figure 9:**
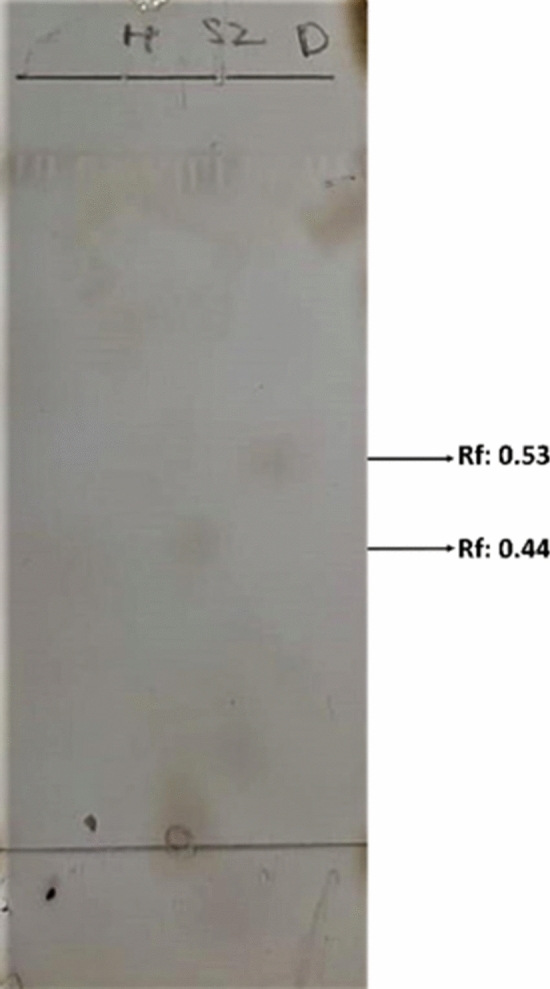
TLC chromatogram of acid-hydrolysed exopolysaccharide from *Nitzschia palea*; showing two different black spots of oligosaccharides derived from the exopolysaccharide of the diatom due to after acid hydrolysis.

After acid-hydrolysis of the exopolysaccharide isolated from the diatom *Nitzschia palea* showing hydrolyzed exopolysaccharide as separate oligosaccharide units in the TLC sheet (Fig. 9). Two different black spots were visible with R*f* values of 0.53, and 0.44.

#### Gas chromatography-mass spectrometry (GC-MS) analysis of monosaccharide composition of the exopolysaccharide isolated from *Nitzschia palea*

**Figure 10:**
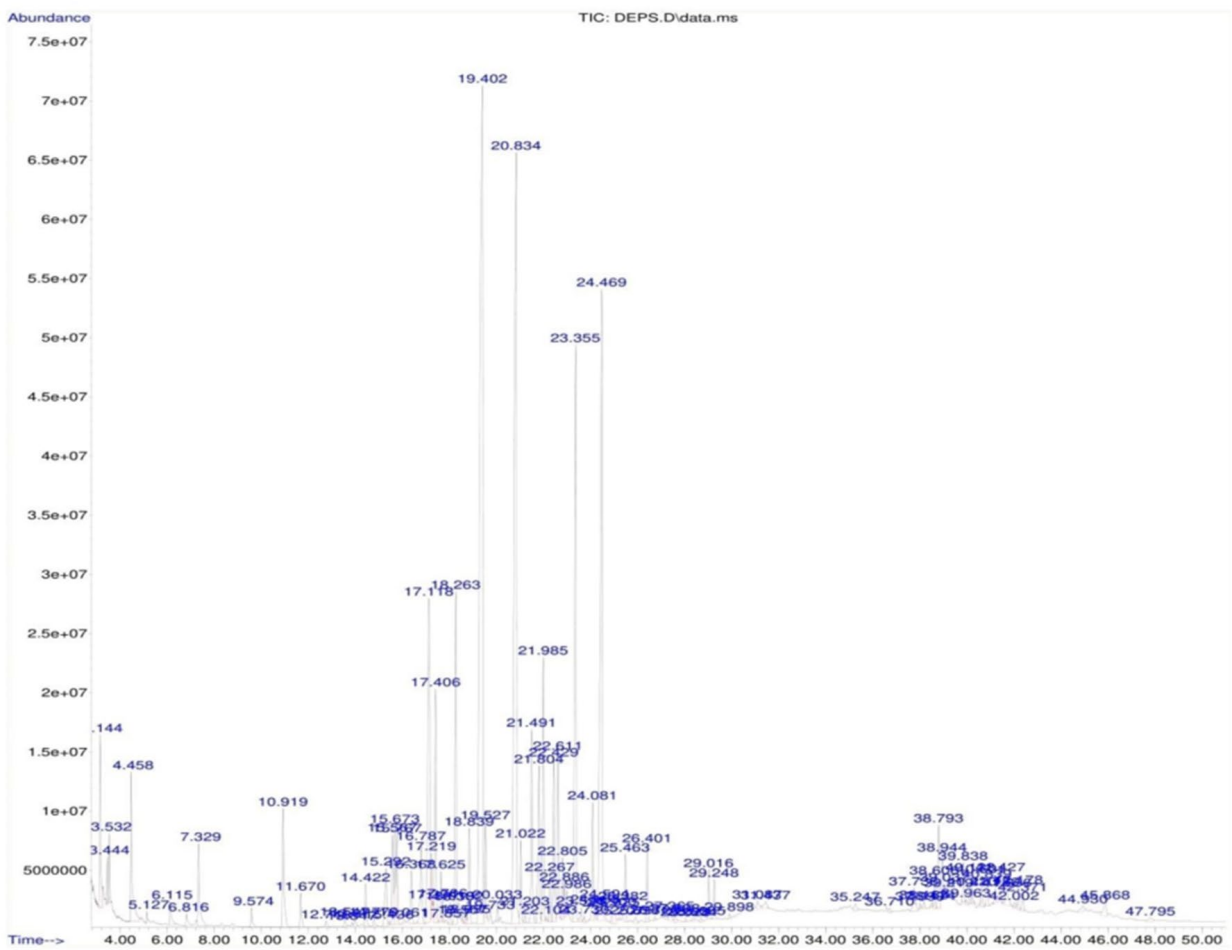
Gas Chromatogram of acid-hydrolysed and derivatised exopolysaccharide of the diatom *Nitzschia palea* showing different monosaccharide units.

**Figure 11:**
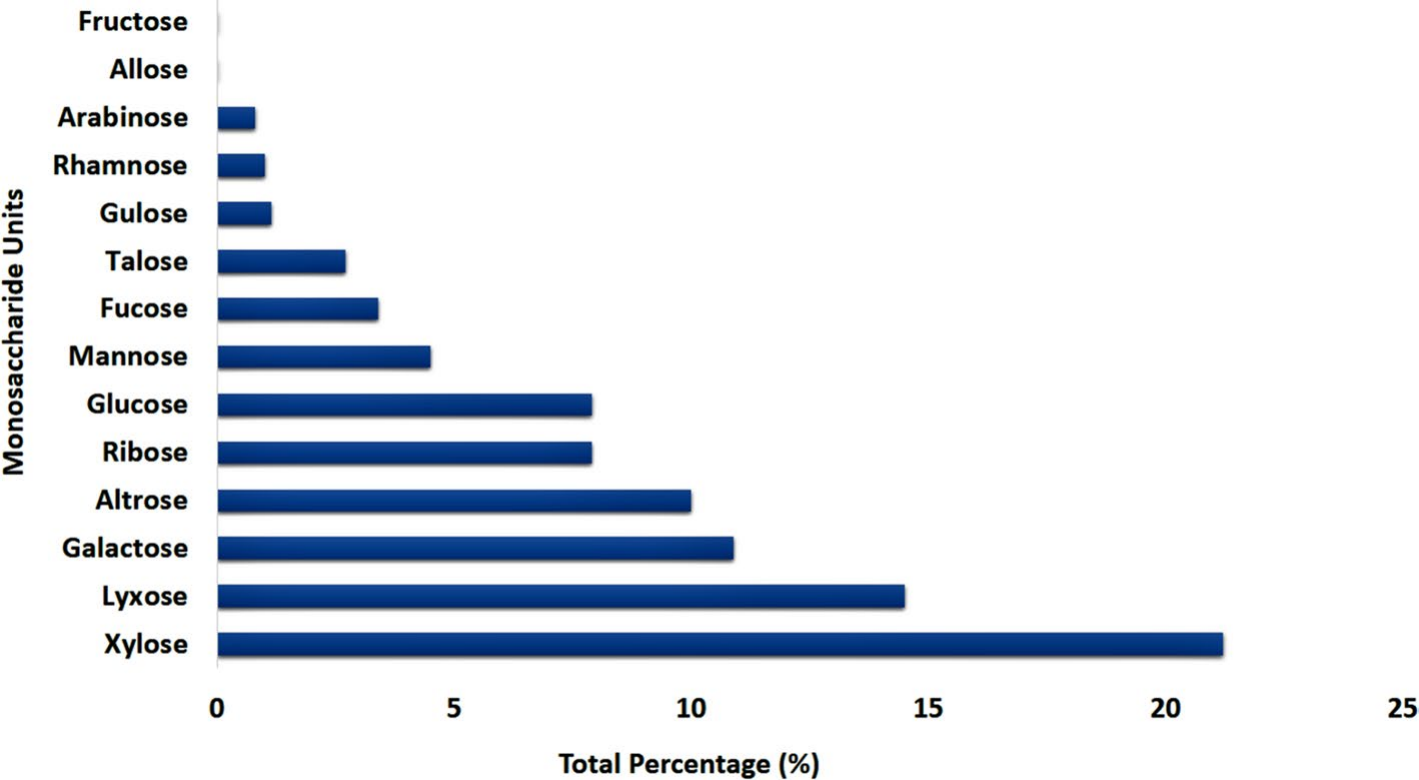
Total percentage of monosaccharide units found in the exopolysaccharide of the diatom *Nitzschia palea* based on GC–MS analysis.

Based on the GC–MS analysis (Fig. 10), it resulted that, xylose (21.16%), lyxose (14.55%), altrose (10.05%), galactose (10.9%), ribose (7.98%), glucose (7.97%), mannose (4.54%), talose (2.72%), gulose (1.14%), rhamnose (1.03%), arabinose (0.78%), allose (0.03%), fructose (0.03%) and fucose (3.45%) were the monosaccharide units being a part of the exopolysaccharide isolated from the diatom *Nitzschia palea* (Table 4). Among them, the top 10 monosaccharide units based on their abundance were xylose, lyxose, galactose, altrose, ribose, glucose, mannose, fucose, talose, and gulose (Fig. 11).

**Table 4:** Monosaccharide composition of exopolysaccharide isolated from the diatom *Nitzschia palea.*

Monosaccharide units	Retention time (RT)	Occurrence percentage (%)	Total percentage (%)
Glucose	13.547	0.106	7.971
	13.847	0.033	
	14.422	0.454	
	15.673	1.012	
	15.767	0.786	
	21.491	1.915	
	21.804	1.811	
	22.886	0.257	
	26.401	0.693	
	36.71	0.06	
	39.037	0.083	
	40.22	0.125	
	40.601	0.305	
	41.427	0.331	
Talose	18.695	0.082	2.722
	24.594	0.099	
	25.382	0.164	
	25.463	0.616	
	38.793	0.859	
	39.838	0.395	
	40.839	0.304	
	42.371	0.203	
Galactose	16.067	0.063	10.902
	21.022	0.753	
	22.429	1.538	
	22.611	1.487	
	23.355	6.393	
	25.025	0.236	
	25.207	0.028	
	37.736	0.404	
Mannose	14.779	0.105	4.546
	19.527	0.736	
	20.033	0.171	
	21.985	2.365	
	24.081	1.1	
	24.193	0.069	
Xylose	15.567	0.989	21.169
	16.368	0.0604	
	17.219	0.394	
	19.402	19.002	
	25.895	0.114	
	39.963	0.066	
Ribose	17.118	5.36	7.985
	17.294	0.025	
	17.406	2.296	
	17.706	0.173	
	18.382	0.131	
Arabinose	17.625	0.49	0.787
	17.857	0.039	
	18.019	0.218	
	26.72	0.04	
Gulose	22.267	0.4	1.146
	38.944	0.443	
	40.138	0.303	
Lyxose	20.834	14.005	14.553
	22.805	0.548	
Altrose	22.986	0.223	10.059
	24.469	9.836	
Rhamnose	18.839	1.038	1.038
Fructose	22.104	0.035	0.035
Allose	28.028	0.032	0.032
Fucose	18.263	3.456	3.456

#### Anticancer potential of exopolysaccharide isolated from *Nitzschia palea*

**Figure 12:**
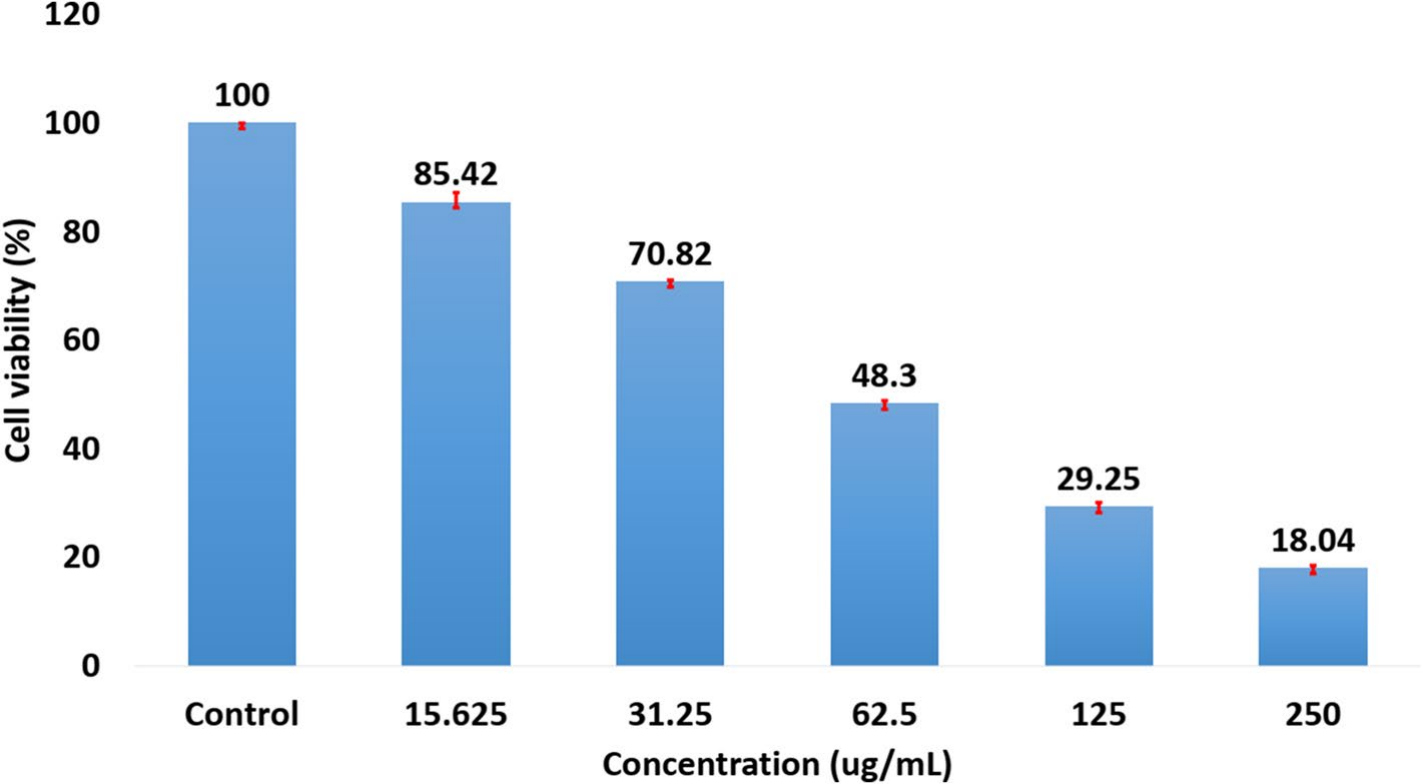
The graph showing decrease in cell viability of Human Lung Adenocarcinoma cells while increasing the concentration of the test sample (Exopolysaccharide isolated from the diatom *Nitzschia palea*).

**Figure 13:**
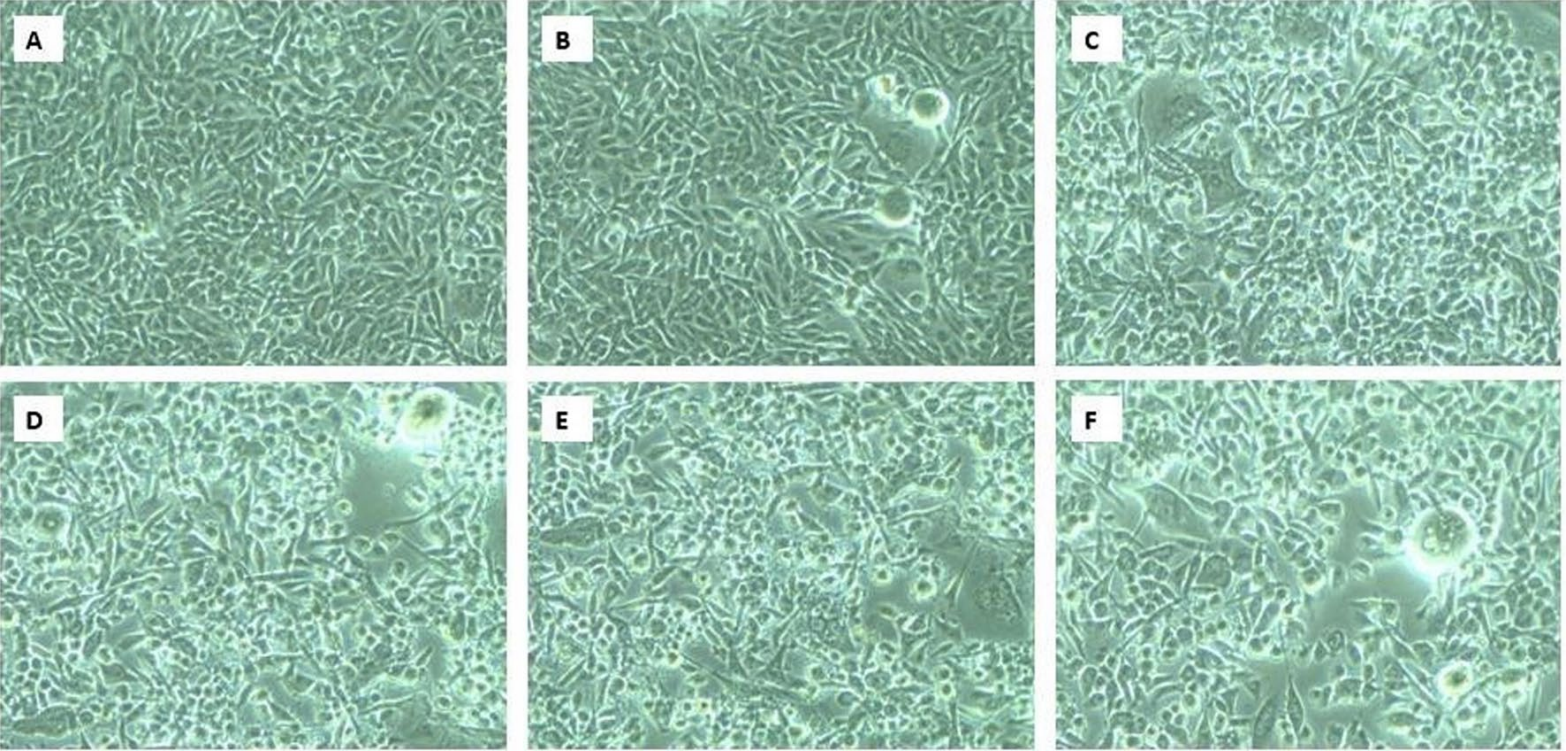
MTT Cell viability assay of the test compound exopolysaccharide isolated from the diatom *Nitzschia palea* on Human Lung Adenocarcinoma; (**A**) Control; (**B**) 15. 625 µg/mL; (**C**) 31.250 µg/mL; (**D**) 62.5 µg/mL; (**E**) 125 µg/mL; (**F**) 250 µg/mL.

The cell viability of human lung adenocarcinoma cells was decreased while increasing the concentration of the test sample (Exopolysaccharide isolated from the diatom *Nitzschia palea*), hence, the concentration of the test sample was inversely proportional to the cell viability of the cancer cells (Fig. 12). Microscopic evaluation also confirms the above results, in which, the number of cells was found decreased while increasing the concentration of the test sample (Fig. 13). Moreover, the estimated IC_50_ value of the test sample (exopolysaccharide from *Nitzschia palea*) was 62.64 µg/mL.

**Figure 14:**
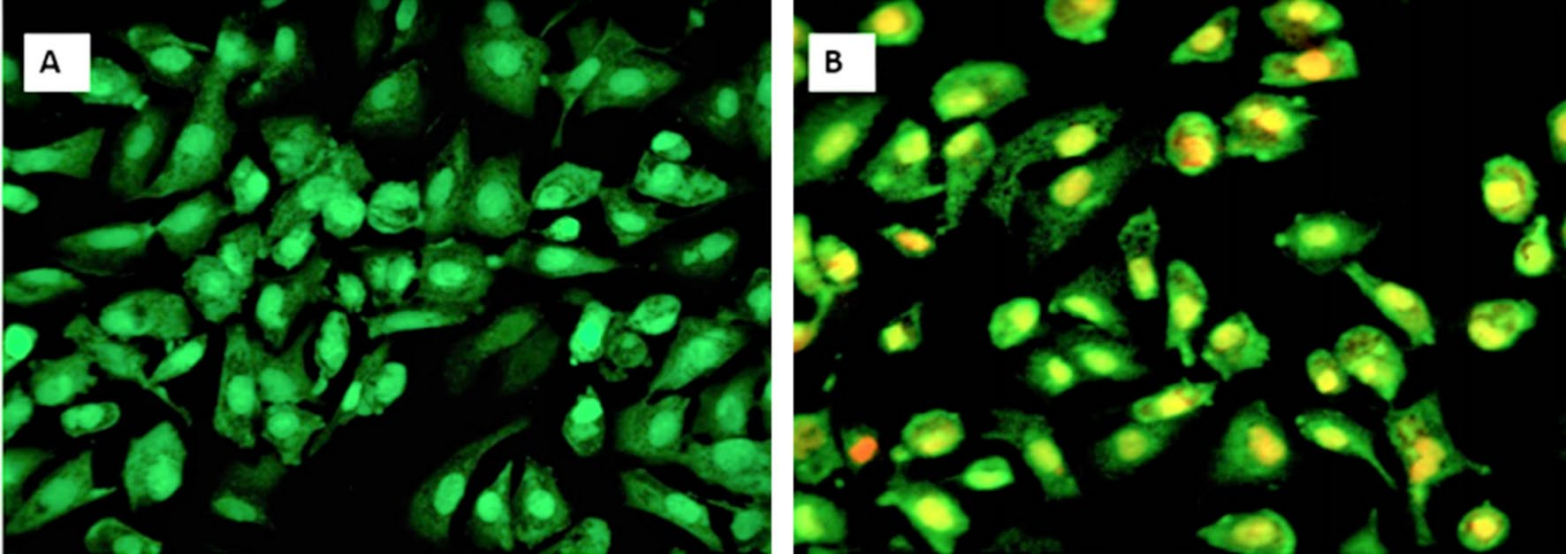
Acridine Orange staining assay; (**A**) Human Lung Adenocarcinoma Control cells; (**B**) Human Lung Adenocarcinoma test sample treated (exopolysaccharide isolated from *Nitzschia palea*).

**Figure 15:**
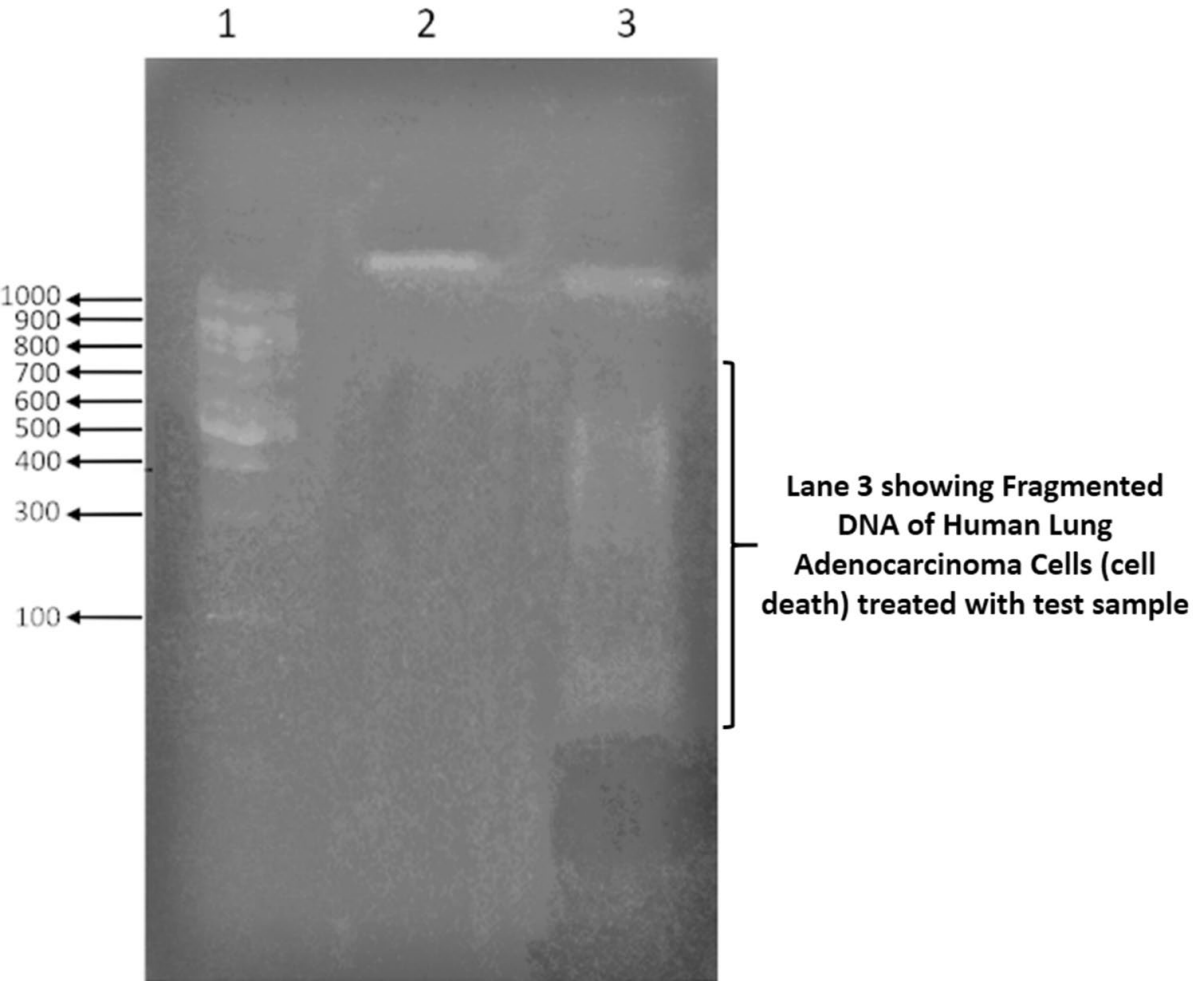
DNA Fragmentation assay; Lane 1: DNA ladder; Lane 2: Human Lung Adenocarcinoma Control Cells showing single DNA band; Lane 3: Human Lung Adenocarcinoma Cells treated with test sample showing fragmented DNA.

The control cells (untreated human lung adenocarcinoma cells), and treated cells (test sample treated human lung adenocarcinoma cells) were stained with acridine orange stain, in which, the control cells were stained with green fluorescence. The treated cells were stained with yellow to bright orange colored fluorescence resulting that the treated cells undergoing apoptosis (Fig. 14). DNA fragmentation assay reveals that unlike control cells (Untreated cells), extracted DNA of the treated cells was found fragmented and dragged as a ladder in agarose gel (Fig. 15) and hence the treated cells underwent apoptosis.

## Discussion

In the present study, the freshwater sample was collected from the inland Chembarambakkam Lake (freshwater) of Kanchipuram District, Tamil Nadu, India. This is a man-made lake built by Emperor Rajendra Chola I (son of Emperor Rajaraja Chola) during the Cholas Era. This lake is one of the rainwater storage reservoirs supplying water to Chennai City other than Puzhal Lake. The physicochemical parameters tested for the collected water sample had shown that the water has optimal conductivity, resistivity, less TDS, and salinity, with high dissolved oxygen content with a pH of 6.1. Therefore, the water is safe for other aquatic flora and fauna and hence, safe to use as drinking water after further treatment.

In this current investigation, for isolation of diatom, a freshwater sample was collected from the Chembarambarambakkam Lake, a PM culture medium was used and the favorable pH was set between 6 and 6.5 as the pH of the water was 6.1. The cosmopolitan dispersion of diatoms in various environments like marine ecosystems, brackish water, and freshwater ecosystems makes them an important primary producer in the food chain, and nutrient cycling in the ecosystem, including several industrial applications^24^. However, the identification of diatoms plays a crucial role in biotechnological applications. Morphological features such as the length, and width of diatoms, including frustule characteristics like striae, fibulae, and raphe are fruitful in the identification of diatoms at species level^25^. Diatom cultures were treated before microscopic studies with acid to obtain clear and transparent frustules as described by Hendey^26^. Therefore, in our study, based on morphological identification, the isolated diatom was identified as *Nitzschia palea*.

Henceforth, due to the wide variety of diatom species, morphological identification is quite challenging and remains problematic to survey the biodiversity of diatoms. To solve this issue, studies on molecular phylogeny were performed for the identification and classification of diatoms. Molecular characterization is a feasible, rapid, accurate, and convenient method for the morphological identification of diatoms. The 18S rRNA gene is the most widely used common gene marker for the molecular identification of microbes^27^. However, the ribulose-1,5-bisphosphate carboxylase large subunit (rbcL) gene from chloroplast was also reported to analyze the phylogeny of diatoms^28,29^. Hence, in the present study, the 18S rRNA smaller subunit gene was selected for molecular characterization of the diatom isolated from the Chembarambakkam Lake, the 285 bp length of 18S RNA smaller subunit partial gene was submitted to NCBI GenBank and the accession number was also retrieved as ON360983. Based on both the morphological and molecular characterization, the isolated pure culture of the diatom was identified as *Nitzschia palea*.

The total carbohydrate content was enhanced under nutrient supply, whereas, hampered when the condition was nutrient scarce and cell density was also found lowered in diatoms^30^. However, nitrogen and phosphate deprivation affect the growth rate of diatom isolated from the Adriatic Sea^31^. Contrastingly, under nutrient deprivation, condition exopolysaccharide synthesis like mannose, glucose, galactose, and uronic acids was found enhanced^32^. In the case of *Nitzschia palea*, (current study) the exponential phase of the diatom was 72–120 h of incubation (3rd–5th day), and the growth rate declined after 144 h (6th day) of incubation. Extraction of polysaccharides can be done with several techniques, in which the most appropriate and effective way of extracting the polysaccharide was done with the procedure of hot water extraction method described by Li and Shah^33^ in which the temperature was changed to 70 °C for only 45 min to avoid interference of any other compounds present in the sample solution. After 12 h of incubation, the exopolysaccharide was precipitated. The total yield of exopolysaccharide content was 1.56 mg of dry weight from 100 mL of PM culture medium inoculated with *Nitzschia palea* on the 7th day of incubation. The estimated carbohydrate content was 51.35 µg/100 µL.

According to Xu et al*.*^34^ the polysaccharide sample showed a distinguished functional group with higher peaks at C = O^24^, which was moreover similar to the FT-IR analysis of the diatom exopolysaccharide showed peaks for C = O and C–O groups denoting the presence of carboxyl groups in our study. The peak near 873 cm^−1^ corresponds to the existence of α-configuration of polysaccharides^34^. Bacterial exopolysaccharide was incubated for 30 min. in HCl and was efficient for complete hydrolysis of exopolysaccharide^35^. But for the eukaryotic exopolysaccharide 60 min. incubation was required for complete hydrolysis. However, in our study, the TLC chromatogram had shown distinct spots of oligosaccharides derived from the exopolysaccharide of *Nitzschia palea*. The optimized mobile phase for TLC was chloroform, acetic acid, and water in the ratio of 6:7:1 reported according to Reiffova^36^.

For GC–MS analysis of polysaccharide, it was hydrolyzed into monosaccharides, and silyated (derivatized) with pyridine, hexamethyldisilazane, and trimethylchlorosilane, which converts monosaccharides into volatile compounds by Ruiz-Matute et al. method^37^. The same protocol was carried out in our study also and found that xylose (21.16%), lyxose (14.55%), altrose (10.05%), galactose (10.9%), ribose (7.98%), glucose (7.97%), mannose (4.54%), talose (2.72%), gulose (1.14%), rhamnose (1.03%), arabinose (0.78%), allose (0.03%), fructose (0.03%) and fucose (3.45%) were the monosaccharide units being a part of the exopolysaccharide isolated from the diatom *Nitzschia palea*. Based on the quantity, xylose, lyxose, galactose, altrose, ribose, glucose, mannose, fucose, talose, and gulose were the top 10 monosaccharide units derived from the exopolysaccharide of *Nitzschia palea*. Urbani et al. (2012) reported rhamnose, fucose, and mannose were the major monosaccharide units that constitute the exopolysaccharide of a marine diatom^38^.

In another study, exopolysaccharide isolated from a bacterium *Bifidobacterium breve* composed of monosaccharide units like rhamnose, arabinose, glucose, galactose, and mannose had shown anticancer activity on head and neck squamous cell carcinoma by promoting apoptosis^39^. Similarly, arabinose-rich exopolysaccharide containing glucuronic acid, and galacturonic acids isolated from *Bacillus* sp. had anticancer potential on HepG2 liver cancer cells with an IC_50_ value of 218 µg/mL^40^. Simultaneously, glucan and mannose-rich exopolysaccharides extracted from *Leuconostoc mesenteroides* were reported to possess anticancer activity on hepatocellular carcinoma^41^. However, the bioactive potential of exopolysaccharides from freshwater diatoms is least studied.

However, Lakshmegowda et al.^42^reported that the hexane extract (volatile compounds) of the freshwater diatom *Nitzschia palea* had shown to possess potent anti-inflammatory activity with an IC_50_ value of 93.5 µg/mL by suppressing proinflammatory cytokines including nitric oxide, IL-6, TNF-α, and PGE2. In this present investigation, the exopolysaccharide extracted from a pennate diatom *Nitzschia palea* was observed to show significant results when treated against human adenocarcinoma lung cancer cell line A549 with an IC_50_ value of 62.64 µg/ml. The mode of its activity was found due to its apoptosis activity was also observed when the cells were viewed under a fluorescence microscope when stained with acridine orange, and DNA fragmentation assay.

Therefore, the exopolysaccharide extracted from the freshwater diatom *Nitzschia palea* isolated from the Chembarambakkam Lake, Tamil Nadu, India found to exhibit potential anticancer activity on Human Adenocarcinoma Lung cancer. This is a *hitherto* report on the anticancer potential of exopolysaccharide from the diatom *Nitzschia palea*.

## Conclusion

A pure isolate of *Nitzschia palea* belongs to the pennate diatom was isolated from the freshwater lake Chembarambakkam, Tamil Nadu, India. Based on the morphological and molecular characterization, it was identified as *Nitzschia palea*. Intriguingly, the exopolysaccharide extracted from the diatom *Nitzschia palea* has potential anticancer activity on the Human Adenocarcinoma lung cancer cell line (A549). Therefore, this diatom exopolysaccharide is a novel bioactive compound with potential anticancer activity.

## Data Availability

The 18S rRNA partial gene of *Nitzschia palea* sequenced was submitted to National Centre for Biotechnology Information (NCBI), and the Accession number retrieved as ON360983 (https://www.ncbi.nlm.nih.gov/nuccore/ON360983.1/**)**.
